# Effectiveness of Pharmacotherapy in Reducing the Inflammation Process of Spinal Cord Injuries: A Systematic Review of Animal Studies

**DOI:** 10.1155/2024/2741367

**Published:** 2024-11-12

**Authors:** Eko Agus Subagio, Pandu Wicaksono, Asadullah Asadullah, Muhammad Faris, Abdul Hafid Bajamal, Imam Susilo, Diaz Syafrie Abdillah

**Affiliations:** ^1^Department of Neurosurgery, Faculty of Medicine, Dr. Soetomo General Academic Hospital, Universitas Airlangga, Surabaya, Indonesia; ^2^Department of Neurosurgery, Brain and Spine Center, Mitra Keluarga Hospital, Surabaya, Indonesia; ^3^Department of Pathology, Faculty of Medicine, Universitas Airlangga, Surabaya, Indonesia; ^4^Faculty of Medicine, Universitas Nahdlatul Ulama Surabaya, Bisui General Hospital, South Halmahera, Indonesia

**Keywords:** inflammatory pathway, pharmacotherapy, spinal cord injury

## Abstract

**Background:** Currently, there is no gold standard technique in SCI therapy. Although there have been many systematic reviews on the pharmacological treatment of inflammation in SCI, there has been no published discussion regarding the effectiveness of anti-inflammatory pharmacotherapy when viewed from a neuroinflammatory pathway. This research aimed to examine an effective and reliable medication for decreasing inflammation in SCI and, where possible, identify effective pharmacotherapeutic treatment protocols.

**Methods:** We searched SCOPUS, PubMed, PlosOne, and Science Direct, for experimental trials published in English. The keywords included (Pharmacotherapy OR pharmacology OR treatment) AND (traumatic spinal cord injury OR spinal cord OR traumatic OR hemisection of spinal cord OR compression OR traumatic spinal cord injury OR aneurysm clip OR hemisection of spinal cord OR sharp pin injury) AND (Side Effect therapy). In addition, the Basso–Beattie–Bresnahan (BBB) score was used to assess post-SCI clinical progress.

**Results:** Twelve pilot studies met the inclusion criteria. The highest BBB score was 15, and the best animal performance was tested after the average therapy was on the second week or 14th day. Three pharmacotherapies have fast effectiveness regarding BBB scores: calcitriol, lithium, and valproic acid. As well as a combination of pharmacotherapy therapy with surgical therapy also get significant results.

**Conclusions:** The preliminary findings showed that many inflammatory pathways cause inflammatory agents to have their pathways for inhibition pathways, so they have different ways.

## 1. Background

The global incidence of spinal cord injury (SCI) is relatively high; around 0.93% of the entire world population (0.78–1.16 million) suffers from SCI each year [1]. Most SCI cases occur in young adult male patients, accounting for 80.7% of all cases with a median age of 28 years. Motor vehicle accidents and falls are two leading causes of SCI [2]. Male individuals in productive age are reported to have the highest risk of SCI due to traumatic events, including individuals whose mean age at the time of the injury ranges between 32 and 55.4 years (in Asia), 37 and 47.9 years (in Europe), and 26.8 and 56.6 (in North America) [3].

There is no gold standard technique in SCI therapy. However, there is a 24-h golden period of therapy for SCI [4]. Reducing inflammation, enhancing SCI pathogenesis, and encouraging nerve regeneration (a reduction in secondary injury) are the goals of future SCI treatment [5]. Methylprednisolone (MP) is now the most common medication to decrease inflammation [6]. Although a high dose of MP therapy may increase the risk of side effects in patients, it is still the mainstay of SCI pharmacological therapy [7]. Some of the most important effects of MP and the rest of the corticosteroids are the result of homeostatic responses by insulin and glucagon. Glucocorticoids stimulate gluconeogenesis, which results in elevated blood glucose, catabolism of muscle protein, and stimulation of insulin secretion. Both lipolysis and lipogenesis become stimulated, with a net increase of fat deposition in certain areas (e.g., face, shoulders, and back) [8].

Numerous research has revealed that the primary goals of SCI treatment strategies, especially for recurrent injuries, are to improve nerve function recovery following SCI and to lessen or stop spinal cord inflammation and apoptosis from accelerating. SCI therapy for neuroprotection focuses on reducing secondary SCI damage, as an important therapeutic target [9].

While there have been many systematic reviews on pharmacological treatment of inflammation in SCI [10], there has been no published discussion of the effectiveness of anti-inflammatory pharmacotherapy from a neuroinflammatory pathway. The most important SCI recovery is to reduce secondary injuries assisted by anti-inflammatories that can reduce inflammatory factors and improve SCI recovery [10].

Since the inflammatory pathways are diverse and each anti-inflammatory has its specificities in SCI conditions, this research aimed to review pharmacotherapy's effectiveness in decreasing inflammation in SCI and, where possible, identify the effective pharmacotherapeutic treatment protocols.

## 2. Methodology

### 2.1. Technique for Search

We searched PubMed, PlosOne, SCOPUS and Science Direct, and Web of Science for experimental trials published in English. Keywords included (Pharmacotherapy OR pharmacology OR treatment) AND (traumatic spinal cord injury OR spinal cord OR traumatic OR hemisection of spinal cord OR compression OR traumatic spinal cord injury OR aneurysm clip OR hemisection of spinal cord OR sharp pin injury) AND (Side Effect therapy). In addition, the exclusion criteria were studies with human trials. Reference lists of included articles were manually searched to identify additional studies. All citations were collected in Mendeley Reference Manager, and duplicates were removed.

**Figure 1:**
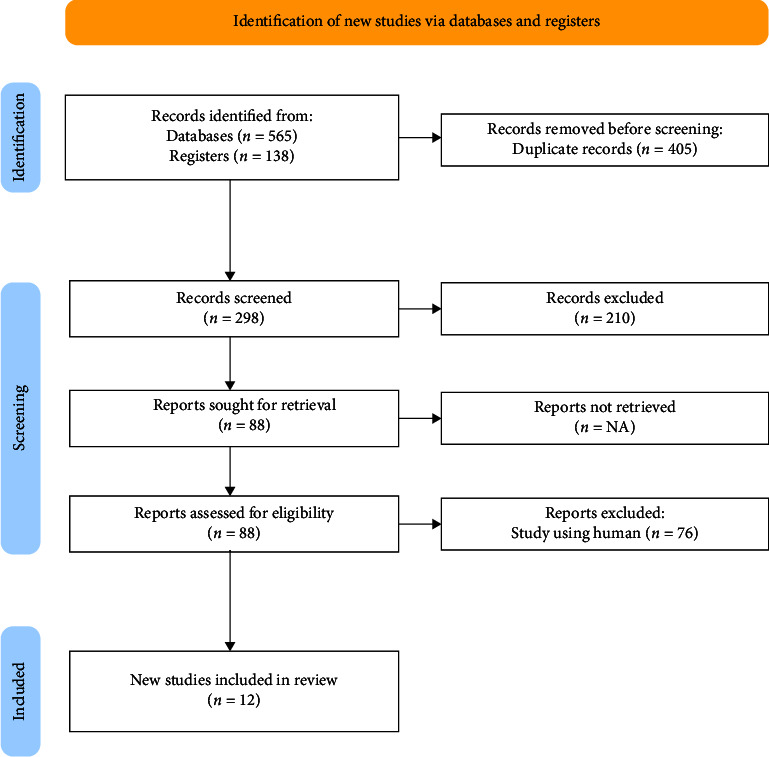
PRISMA 2020 flow diagram.

This systematic review was compliant with the Preferred Reporting Items for Systematic Reviews and Meta-analysis (PRISMA) 2020 statement [11]. The PRISMA guideline is commonly used for clinical trials, but we adapted it for this systematic review [12]. The inquiry was conducted in May 2024. The summary of the search strategy is displayed in Figure 1.

### 2.2. Study Selection

Articles published in English employing pharmacotherapy as a treatment for SCI, categorized as experimental trials using animal trials with a spinal cord injury, were included. We excluded any study using humans. The first author was responsible to minimize subjective bias and review all articles that fit the criteria. Meanwhile, other authors were responsible to check the title and abstract as a preliminary study. The content of the research papers was evaluated if their title and abstract suited the criteria. The third reviewer discussed further clarifications or disputes from the published articles. Articles included in the review process must be a pharmacotherapeutic clinical trial study of SCI evaluating the inflammatory process. Those with a high risk of bias were omitted, including studies in which the effect of pharmacotherapy on the prevention of SCI was the major outcome of interest.

A systematic literature search was conducted to identify studies in animal models. This systematic review composed the question research based on PICOS: P—population; I—intervention; C—comparative interventions; O—outcomes; and S—type of study. For the question “What is the effectiveness of pharmacotherapy in reducing inflammation in SCI in experimental animal models?” the PICOS was P—animal models (rats, mice); I—injury spinal cord to animal model; C—give treatment pharmacotherapy; O—Inhibition inflammatory in SCI rats; and S—experimental studies.

### 2.3. Data Extraction and Synthesis

Several information was extracted: (1) author and year of publication, (2) sample size, (3) type of experimental animal, (4) SCI location, and (5) Scopus qualification (see Table 1). Outcome measures were listed, the mean and standard deviation was also recorded for participant groups, and the major results for each study are listed in Table 2.

### 2.4. Methodological Quality

We assessed the quality of each individual study using the 10-item checklist of CAMARADES. The criteria comprise (1) publication in a peer-reviewed journal, (2) statement of control of temperature, (3) randomization to treatment or control, (4) blinded induction of SE (i.e., concealment of treatment group allocation at the time of induction of SE), (5) blinded assessment of outcome, (6) a measure of trainability and inclusion of scale 3 or above animals, (7) adaptation/familiarization to exercise apparatus, (8) sample size calculation, (9) statement of compliance with regulatory requirements, and (10) statement regarding possible conflicts of interest [13].

## 3. Result

### 3.1. Study Selection

Figure 1 shows the article selection method PRISMA 2020 [11]. A comprehensive search in PubMed (310 articles), PlosOne (255 articles), and Science Direct (138 articles) generated a total of 703 publications. Two hundred ninety-eight articles were evaluated after removing duplicates. Then, 210 articles were removed because the abstract did not comply with the inclusion criteria, leaving 88 articles for further analysis. However, 76 articles were excluded because SCI individuals had other neurological problems and the study did not use an experimental design, leaving 12 articles compliant with the predetermined criteria for further review.

### 3.2. Quality of Methodology

The median range quality score for the 12 included studies was 8.00 ± 1.23 (range 3). All articles were published in peer-reviewed journals. All articles do not mention control of temperature during injury to rats, and random allocation to groups was reported in all included studies. Allocation concealment was reported in 9 of 12 studies, whereas blinded assessment was documented in 7 of 12 studies. A measure of trainability and inclusion of scale 3 or above animals was reported in all included studies whereas the use of adaptation and familiarization to exercise apparatus was described in 11 of 12 studies. Performing a sample size calculation was not documented in all included studies, a statement of compliance with regulatory requirements was reported in all included studies, and a statement of conflicts of interest was reported in 5 of 12 studies (Table 2).

### 3.3. Description of Included Studies

#### 3.3.1. Animal Model of Acute Spinal Cord Injury

Twelve reviewed studies used a total of 1150 experimental animals with SCI. Sample sizes ranged considerably from 45 to 80 animals. The majority of the experimental animals were male Sprague–Dawley (SD) rats.

There were two methods used to induce injury in these 12 studies. First, researchers performed a laminectomy on the vertebrae to open the spinal cord, mostly on vertebrae T9-T10, and induced injury by dropping objects (metal rods of 20–30 g) above the exposed spinal cord (10–25 mm). Second, researchers exposed the spinal cord and clamped it using vascular clips with varying force and duration to induce spinal injury.

### 3.4. Outcome Measurement

The Basso–Beattie–Bresnahan (BBB) score, ranging between 0 (total paralysis) and 21 (normal locomotory function), was employed to assess functional outcome following SCI [14] (Table 3).

## 4. Adverse Effects or Side Effects

The chosen investigations reported no adverse effects, side effects, or hazardous complications in experimental animals.

## 5. Discussion

### 5.1. Effective Alternative Pharmacotherapy

Each anti-inflammatory agent has its peculiarities because each has a different pathway but has the same goal: to reduce inflammation. It is critical to note that secondary injury in SCI activates damage-associated molecular patterns (DAMPs). At the macrophage level, DAMP binds to the toll-like receptor (TLR) on the cell membrane, increasing the activity of NF-kB via M1 activation. NF-kB stimulates the inflammatory response by affecting proinflammatory genes in the cell nucleus, namely, TNF and IL-1. TNF via tumor necrosis factor receptor-2 (TNFR-2) will activate the caspase cascade via the Fas-associated death domain (FADD). Caspases are released into the cytoplasm, causing cells to die [25].

In a study by Khajoueinejad et al., calcitriol (1-alpha-dihydroxyvitamin D3) was used. It is generally known as a compound related to calcium homeostasis and bone health, but along with technological developments, it affect calcitriol on immnune system on immune system. The therapeutic target of calcitriol is lymphocytes. Other data sources show that calcitriol can inhibit the production of IFN-*γ* by peripheral blood mononuclear cells. The results of Khajoueinejad's et al. research showed the same thing that administration of calcitriol in the acute phase after SCI can result in a decrease in IFN-*γ* secretion in mononuclear cells and suppress the production of other proinflammatory cytokines such as IL-17. So it is useful in improving the recovery of motor function [16].

In Wang et al. study, lithium promotes the production and release of neurotrophins, stimulates neurogenesis, enhances autophagy, and inhibits apoptosis. In addition, lithium can be used to protect neurons after SCI and reduce inflammation after injury. The results of research by Wang et al. show that administering lithium to SCI rats reduced apoptosis by the upregulation of antiapoptotic molecule (Bcl-2) expression and the decline of proapoptotic factor (Bax and cleaved caspase-3) expression and alleviated the increase of proinflammatory cytokine (including TNF-*α*, IL-6, and IL-1*β*) secretions by inactivating the NF-*κ*B pathway in SCI rats [14].

In Chen's et al. research, valproic acid, commonly used to treat epilepsy, was used. According to recent research, valproic acid is a histone deacetylase inhibitor of class 1/II (HDCA), which inhibits the actions of HDAC. Acetylated histone proteins protect neurons by decreasing inflammation and suppressing neuronal death. HDCAi appears to limit NF-kB transcriptional activity by keeping NF-kB acetylated (inactive) and suppressing the inflammatory response. NF-kB is regarded as an inflammatory mediators' main transcription factor, essential for microglial activation. In Chen's research, it was stated that the administration of valproic acid to experimental animals produced an anti-inflammatory response by modulating microglia polarization through STAT1-mediated acetylation of the NF-kB pathway [21].

Other research reported the use of other pharmacotherapies such as the agonist rosiglitazone, and JQ 1 (anti-inflammatory reagent) has negatively modulated inflammation and inhibits BRD4 on inflammatory response in SCI [19]. Other research has almost the same mechanism in the inflammatory response by inhibiting inflammatory factors such as the NF-*κ*B, IL-1, and TNF-*α* pathways, among others, and even reducing apoptosis in experimental animals, the following are types of pharmacotherapy from previous studies agonist rosiglitazone [20], paeonol [17], curcumin [15], glycerol triacetate [18], metformin [9], gastrodin [26], epifriedelinol [27], sparstolonin B [23], and selenium [28].

In SCI conditions, it is pivotal to consider secondary SCI. In this regard, the pathophysiological mechanism of secondary injury is always a concern for researchers. Secondary SCI has long been considered a critical therapeutic target for neuroprotection and functional maintenance in SCI therapy [8]. In addition, the SCI therapy also focuses on preventing apoptosis in the spinal cord and accelerates recovery and condition of nerve function after SCI [5, 23].

In research Assadullah's study on the prevention of apoptosis, adrenocorticotropic hormone compounds 4–10 (ACTH4-10) or ACTH (4–10) Pro8-Gly9-Pro10 is reported to modulate inflammation in mild to severe SCI, reduce secondary injury, and can prevent apoptosis. In recent studies, ACTH/MSH exposure (4–10) significantly reduced caspase-9-producing cells in mild and severe SCI. Caspase-9 is an important compound in the mechanism of apoptosis [26].

This phase of acute secondary injury lasts from 2 to 48 h. Continuous bleeding, edema, and stages of inflammation lead to substantial necrosis as indicated by increased concentrations of specific inflammatory agents and the presence of structural biomarkers, such as IL-7 in the cerebrospinal fluid (CSF) [29].

Our findings demonstrated that intraperitoneal calcitriol administration in acute phase post-SCI could enhance motor function recovery with reduced IL-17 proinflammatory cytokine production and have no significant effects on IL-10 as an anti-inflammatory cytokine. IL-17 is an important proinflammatory factor mainly produced by Th17 cells, contributing to neuroinflammation and disease pathogenesis in most autoimmune diseases. Calcitriol has been reported to decrease oxidative stress in rats at day-7 post-SCI. Furthermore, that calcitriol treatment in the acute phase after SCI could result in decreased IFN-*γ* secretion in isolated mononuclear cells against MBP, whereas IL-4 production could be increased [16].

### 5.2. Effectiveness of Treatment: BBB Scale

Regarding the BBB scale, many researchers conducted the weekly follow-up until the week-12. Some studies evaluated the result once after the therapeutic intervention was done. Comparing single evaluation to weekly evaluation, the latter exhibited better performance in examining changes and in finding out the period of the significant recovery.

The review results show (Table 3) that the 12 articles have their own timeline to find out the best recovery in experimental animals. Each type of treatment has a best time for evaluating recovery from the BBB score. According to research conducted by Khajoueinejad et al., the investigation found that the fastest recovery was recorded that uses calcitriol. Their study reported a BBB score of 12 on the 14^th^ day or the second week, which was a significant increase from the first week and was closer to the normal score. The study also made a statistical comparison to the SCI without treatment group (*p* < 0.05) [16].

Another interesting finding was noticed in Zhang et al.'s research that used metformin. Similar to Calcitriol, the use of metformin was reported to achieve a BBB score of 14 in the second week, significantly increasing from a score of 8 in the first week with a *p* value < 0.01. The study also compared results between metformin and MP therapy and found that the former exhibited better performance, although not significant (*p* > 0.05) [30].

A gradual increase was noticed in each follow-up evaluation in Wang et al. study, showing that JQ1 may inhibit BRD4 as an inflammatory agent. JQ1, also known as thienotriazolodiazepine, is an effective inhibitor of the BET family of bromodomain proteins, which includes BRD2, BRD3, and BRD4, where the latter is found to BRD4 influence SCI inflammation. Based on the BBB scale, the results of the 3-week investigation showed a slow, constant increase every week with a score of 13 on the 21st day. In fact, JQ1 is useful and functions as one of the SCI therapies. However, taking other aspects into consideration, JQ1's low availability is one of the drawbacks, in addition to its expensive price. Thus, the choice regarding JQ1 can be used as an additional reference for research but is difficult to apply in daily practice [19].

In a study by Guo et al. regarding N-acetylcysteine (NAC), it was found that the evaluation of the development of therapy was very long, which took up to 60 days or approximately 2 months. The BBB score was evaluated every 10 days and the 60th day recorded the best score. In this study, early decompression surgery (8 hours post-SCI) was compared to late decompression surgery (48 hours post-SCI). Then, NAC was given after decompression, and the results of the BBB score for early decompression surgery with NAC were better than late decompression surgery. Moreover, the result reduces SCI-induced nerve damage, inflammatory response, and apoptosis, as indicated by increased myelinated nerve area.

The study also emphasized that early decompression combined with NAC would inhibit apoptosis and improve neurological outcomes. Thus, NAC is reported to have a neuroprotective effect by inhibiting inflammatory and apoptotic responses [24].

### 5.3. The Primary Pharmacotherapy for SCI: MP

In several studies, MP has become one of the main options for treating SCI, especially during the secondary phase of SCI. MP may decrease inflammation by reducing T-cell activation and extravasation [31]. Most SCI therapies focus on reducing secondary injuries such as excitotoxicity, lipid peroxidation, and inflammation. In late 2017, AOSpine guidelines said glucocorticoid is not a standard treatment but suggested the use of high doses of MP. American Academy of Emergency Medicine states that it is an acceptable option [32].

However, since there has been no standard treatment in SCI and many complications are reported in long-term use of MP, many researchers have found several other alternative pharmacological therapies [5].

## 6. Conclusion

This study systematically reviewed 12 journal articles that met the inclusion criteria and shared the same purpose: to reduce inflammation in SCI. Many inflammatory pathways create various mechanisms of inhibitory pathways, leading to different effects. The speed of recovery factor in SCI experimental animals is also influenced by several factors. In addition to the effectiveness of each pharmacotherapy, the degree of injury to the spinal cord in each article also varied. Phase of acute secondary injury lasts from 2 to 48 h, which is an important phase in SCI management. This systematic review is expected to be useful for further experimental research and can be used as a reference regarding the effectiveness of pharmacotherapy in SCI.

## Figures and Tables

**Table 1:** Characteristics of selected studies.

Author/year	Number of experiment	Intervention	Type sample experimental/location of SCI	Process injury	Function/result	Efficacy of procedure	Scopus
Zhang et al. 2017	*n* = 100	Curcumin treatment	Rat model (female)/T9–T11	The force was applied via a stainless steel rod 3 g	Inhibition of inflammation and TAK1 pathway	Significant	Q2
Khajoueinejad et al. 2019	*n* = 36	Immunomodulatory effects of calcitriol	Rat model (male and female)/T8–T11	10 g weight (a 2 mm metal rod) from 12.5 mm height centered above exposed spinal cord	Significant reduction in IFN-*γ* and IL-17A secretion and leukocyte infiltration into injury site	Significant	Q1
Liu et al. 2020	*n* = 268	Targeting protein deacetylation (glycerol triacetate (GTA) treatment)	Rat model (male and female)/T8	Injury by dropping a 30 g weight from a height of 5 cm onto the exposed spinal cord	The results of TUNEL–NeuN double staining showed that GTA markedly reduced the number of apoptotic neurons in the SCI group. In addition, the production of inflammatory cytokines TNF-*α*, IL-1*β*, and IL-6 was also suppressed after treatment of GTA compared with SCI rats	Significant	Q2
Zhang et al. 2020	*n* = 90	Therapeutic effect of metformin	Rat model (male)/T9-T10	Injury by dropping a 10 g; diameter of 2 mm was dropped from a height of 30 mm	Increased the expressions of *β*-catenin and brain-derived neurotrophic factor (BDNF), inhibited neuron apoptosis and inflammatory response	Significant	Q2
Wang et al. 2021	*n* = 30	Lithium alleviated	Rat model (male)/T9–T12	Injury by dropping using a 10 g rod dropped from a height of 10 mm	Lithium reduced SCI-induced apoptosis and inflammation in rats	Significant	Q1
Wang et al. 2019	*n* = 30	BRD4 inhibition attenuates inflammatory response in microglia	Rat model (female)/T9-T10	Injury by clamped using a vascular clip (10 N force) for 60 s	BRD4 inhibition by JQ1 not only blocked microglial M1 polarization but also repressed the level of proinflammatory cytokines in microglia in	Significant	Q2
Li et al. 2013	*n* = 36	Peroxisome proliferator-activated receptor-*γ* agonist rosiglitazone reduces secondary damage	Rat model (male)/T6-T7	Injury by an aneurysm clip (closing force 24 g)	Agonist rosiglitazone may be useful in the treatment of SCI. Reduced levels of TNFa and IL-1b, MPO activity, and apoptosis in the injured spinal cord		Q3
Chen et al. 2018	*N* = 192	Valproic acid attenuates traumatic spinal cord injury-induced inflammation	Rat model (male)/T10	Injury (10 g × 25 mm) using a 10 g metal rod dropped from a height of 25 mm	These results suggested that the valproate acid treatment attenuated the inflammatory response by modulating microglia polarization through STAT1-mediated acetylation of the NF-*κ*B pathway	Significant	Q1
Zhao et al. 2022	*N* = 48	Paeonol regulates NLRP3 inflammasomes	Rat model (female)/T9	Injury by vascular clip (30 g forces) for 60 s	Paeonol reduced the levels of apoptosis-associated speck-like protein (ASC), NLRP3, active caspase 1, and N-gasdermin D (N-GSDMD) and repressed the contents of IL-1*β*, IL-18, TNF-*α*, and MDA and elevated GSH level	Significant	Q2
Du 2016	*N* = 60	Gastrodin (gas) ameliorates spinal cord injury via antioxidant and anti-inflammatory effects	Rat model (male)/T8-T9	Injury (10 g × 25 mm) using a 10 g metal rod dropped from a height 25 mm	GAS may promote the recovery of SCI through the enhancement of nrf2-GCLc/GCLm signaling pathway, and subsequent improvement of oxidative stress and inflammation	Significant	Q2
Yuan et al. 2016	*N* = 30	Sparstolonin B attenuates spinal cord injury-induced inflammation in rats by modulating TLR4-trafficking	Rat model (male)/T10	Injury by vascular clip (no data forces) for 30 s	Sparstolonin B may attenuate SCI‐induced inflammation and apoptosis in rats	Significant	Q3
Guo et al. 2022	*N* = 150	N-acetylcysteine alleviates spinal cord injury in rats after early decompression surgery by regulating inflammation	Rat model (female)/T9	Injury by compression by a platform (area 2.5 mm, then a weight of 50 g, for 5 min	NAC could be a promising combination for the treatment of acute SCI, and its therapeutic effects may be associated with the regulation of inflammation and apoptosis	Significant	Q2

**Table 2:** Quality checked on The CAMARADES checklist.

Author/year	1	2	3	4	5	6	7	8	9	10	Quality score
Zhang et al. 2017	Y	NM	Y	NM	NM	Y	Y	Y	Y	NM	6
Khajoueinejad et al. 2019	Y	NM	Y	NM	Y	Y	Y	Y	Y	Y	8
Liu et al. 2020	Y	NM	Y	Y	Y	Y	Y	Y	Y	Y	9
Zhang et al. 2020	Y	NM	Y	Y	Y	Y	Y	Y	Y	Y	9
Wang et al. 2021	Y	NM	Y	Y	Y	Y	Y	Y	Y	Y	9
Wang et al. 2019	Y	NM	Y	Y	NM	Y	NM	Y	Y	NM	6
Li et al. 2013	Y	NM	Y	Y	NM	Y	Y	Y	Y	NM	7
Chen et al. 2018	Y	NM	Y	Y	Y	Y	Y	Y	Y	NM	8
Zhao et al. 2022	Y	NM	Y	Y	Y	Y	Y	Y	Y	Y	9
Du 2016	Y	NM	Y	Y	NM	Y	Y	Y	Y	NM	7
Yuan et al. 2016	Y	NM	Y	NM	NM	Y	Y	Y	Y	NM	6
Guo et al. 2022	Y	NM	Y	Y	Y	Y	Y	Y	Y	NM	8

Abbreviations: NM = not mentioned, Y = yes.

**Table 3:** Outcome measures.

Reference	Outcome measure	Assessment time	Experimental group	Results
Zhang et al. [15]	BBB	The locomotor recovery was evaluated at 1, 7, 14, 21, and 28 days following the SCI.	0 4 7 11 14 *p* < 0.05 compared with SCI no treatment	All injured mice were almost completely paraplegic on the first day, and starting on day 14, the BBB score showed significant results
Khajoueinejad et al. [16]	BBB	The rats were tested 2, 7 days after SCI, after which their assessment was performed once a week until 12 weeks after SCI by observers blinded to experimental treatment	0 9 12 12,5 13 13 13 12 13 13 13 13 *p* < 0.05 compared with SCI no treatment	Statistical analysis revealed that the BBB score in the calcitriol treatment group starting from the second week showed a significant increase in progress compared to the SCI control group
Liu et al. [17]	BBB	The locomotor recovery was evaluated just once after 2 weeks of treatment	16 *p* < 0.0142	Treatment with GTA significantly improved the BBB scores of the SCI group
Zhang et al. [18]	BBB	The BBB scores were utilized to determine locomotor functional recovery before surgery and 1, 3, 7, 14, 21, and 28 days after SCI	0 5 8 14 15 15 *p* < 0.01 compared with SCI no treatment	The BBB scores of the rats in the metformin and methylprednisolone groups were evidently higher than those of the rats in the SCI group after 14 days. There was no statistically significant between the metformin group and methylprednisolone group at each time point (*p* > 0.05)
Wang et al. [14]	BBB	The locomotor recovery was evaluated at 1, 7, 14, and 21 days following the SCI	4 7 11 12 *p* < 0.05 compared with SCI no treatment	The BBB scores of the lithium treatment group continued to increase compared to the SCI group
Wang et al. [19]	BBB	The locomotor recovery was evaluated at 1, 5, 7, 14, and 21 days following the SCI.	0 5 7 10 13 *p* < 0.01 compared with SCI no treatment	The results of BBB scores showed that SCI rat with no treatment displayed a lower functional recovery rate and maximum lower scores compared to those with JQ1 treatment after injury
Liu et al. [20]	BBB	The locomotor recovery was evaluated at 3, 7, 10, 14, and 21 days following the SCI.	2.15 4.52 6.25 8.46 10.22 *p* < 0.05 compared with SCI no treatment	Rosiglitazone treatment resulting in significantly better motor function compared with animals in the SCI
Chen et al. [21]	BBB	The locomotor recovery was evaluated at 1, 3, 7, and 14 days following the SCI	0 3 8.17 12.6 The neurological functions were severely impaired immediately after the SCI (*p* < 0.05)	The BBB scores of these animals gradually returned to the control values, with significant improvement observed in the rats on day 7
Zhao et al. [17]	BBB	The locomotion function of rat after SCI at the time point of 1, 3, 7, 14, and 21 days	0 3 7 10 12 *p* < 0.001 versus the SCI + CMC-Na group.	Paeonol treatment resulting in significantly better motor function compared with animals in the SCI and with SCI and CMC-Na
Du et al. [22]	BBB	The locomotor recovery was evaluated at 1, 3, 7, 21, and 28 days following the SCI	0 5 6 7 10 14 *p* < 0.05 compared with SCI no treatment	BBB scores in rats of SCI group slowly increased during 1–28 days, indicating that locomotor functions of rats were gradually recovered
Yuan et al. [23]	BBB	The locomotor recovery was evaluated just once after 4 weeks treatment	14 *p* < 0.01 compared with SCI no treatment	Treatment with sparstolonin B significantly recovered
Guo et al. [24]	BBB	The locomotor recovery was evaluated at 0, 10, 20, 30, 40, 50, and 60 days following the SCI	0 4 6 9 11 12 13 *p* < 0.05 compared with SCI no treatment	Compared with the DEC groups, the BBB scores in the corresponding DEC + NAC combination treatment groups increased

## Data Availability

All data of this manuscript are included in this main manuscript.
